# Understanding Palmar Fasciitis and Polyarthritis Syndrome as a Rheumatologic Paraneoplastic Syndrome: A Case Report

**DOI:** 10.7759/cureus.61248

**Published:** 2024-05-28

**Authors:** Blessing Eze, Mark Freijat

**Affiliations:** 1 Internal Medicine, Creighton University School of Medicine, St. Joseph's Hospital and Medical Center, Phoenix, USA; 2 Rheumatology, Flow Rheumatology, Phoenix, USA

**Keywords:** palmar images, rheumatologic process, paraneoplastic syndrome, malignant neoplasms, palmar fasciitis and polyarthritis syndrome

## Abstract

Palmar fasciitis and polyarthritis syndrome (PFPAS) is an exceedingly rare rheumatologic condition characterized by fibrotic changes in the palmar fascia with joint pains. It is known to be associated with gynecological malignancy, especially ovarian adenocarcinoma, gastric cancer, pancreatic, prostate, breast, and lung cancer. We present a unique case of a 75-year-old Caucasian female with PFPAS preceding the diagnosis of ovarian cancer by eight months.

Our case highlights the importance of considering PFPAS as a potential paraneoplastic syndrome. It underscores the need for increased awareness and further studies to enhance the early detection of underlying malignancies in patients presenting with similar nonspecific hand symptoms.

## Introduction

Palmar fasciitis and polyarthritis syndrome (PFPAS) is a rare paraneoplastic syndrome originally described by Medsger et al. in 1982, a condition characterized by inflammation of the palmar fascia, tendon sheaths, palmar thickening, generalized arthritis of the small joints of the fingers and wrist that leads to fibrotic changes, and rapid development of flexion contractures in the hands and wrists [1-9]. Since the initial description, there have been 48 case reports in the PubMed search engine, with only 9 occurring in the last decade. More than 69% have ovarian-related cancer [1,2]. The timing of the appearance of PFPAS to malignancy varies; amongst 48 reported case reports, 50% of the PFPAS preceded the underlying malignancy with an average of 6 months; others were reported after diagnosis and during treatment of advanced malignancy [8-9].

Herein, we describe a case where PFPAS preceded the diagnosis of serous ovarian cancer by eight months. This case underlines the intricacy of a patient with multisystemic symptoms, found to have a major malignancy, and responding positively to targeted therapy.

## Case presentation

**Figure 1:**
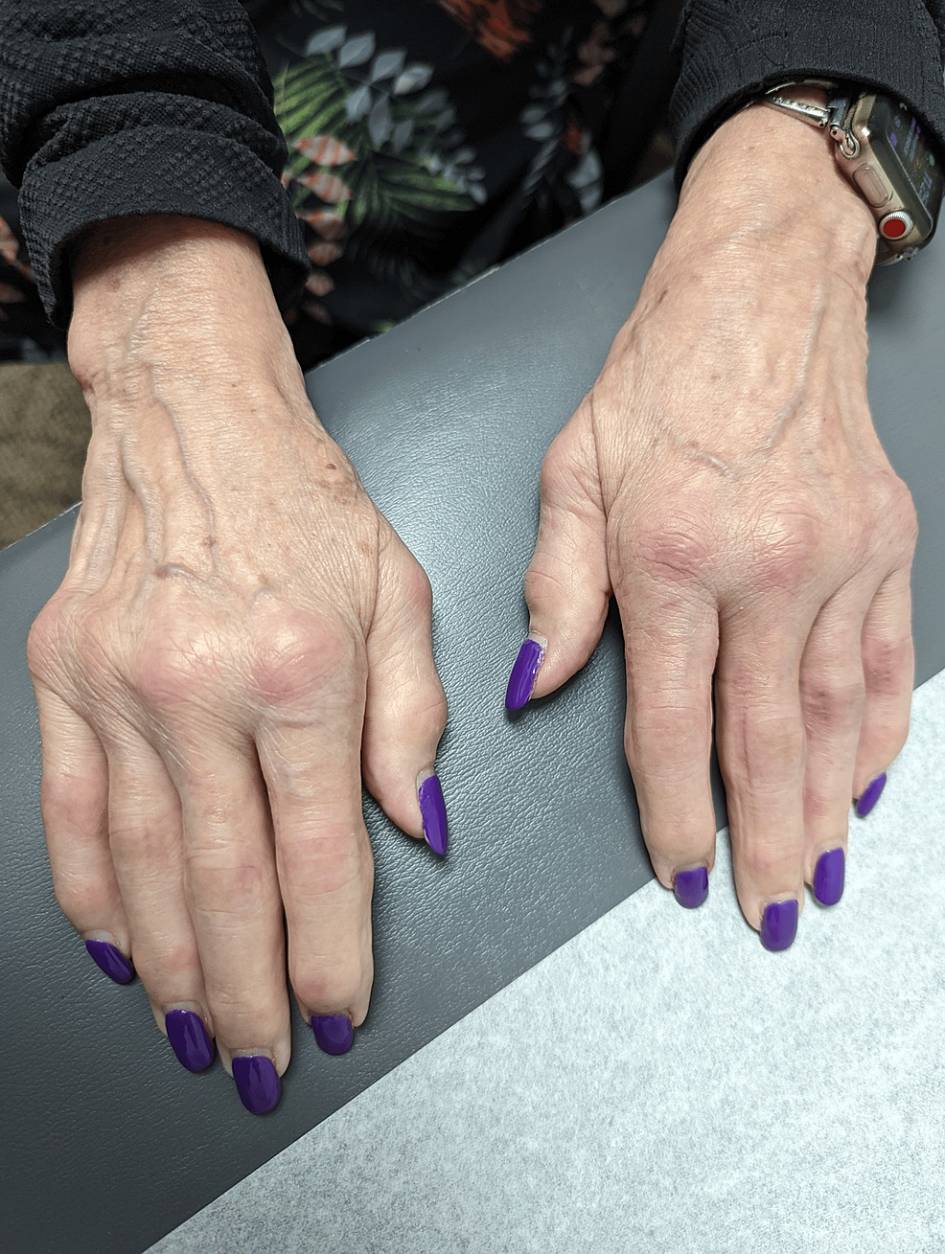
PIP, DIP, and MCP with erythema in the presence of nodular-appearing joints​ PIP: proximal interphalangeal joint, DIP: distal interphalangeal joint, MCP: metacarpophalangeal joint

**Figure 2:**
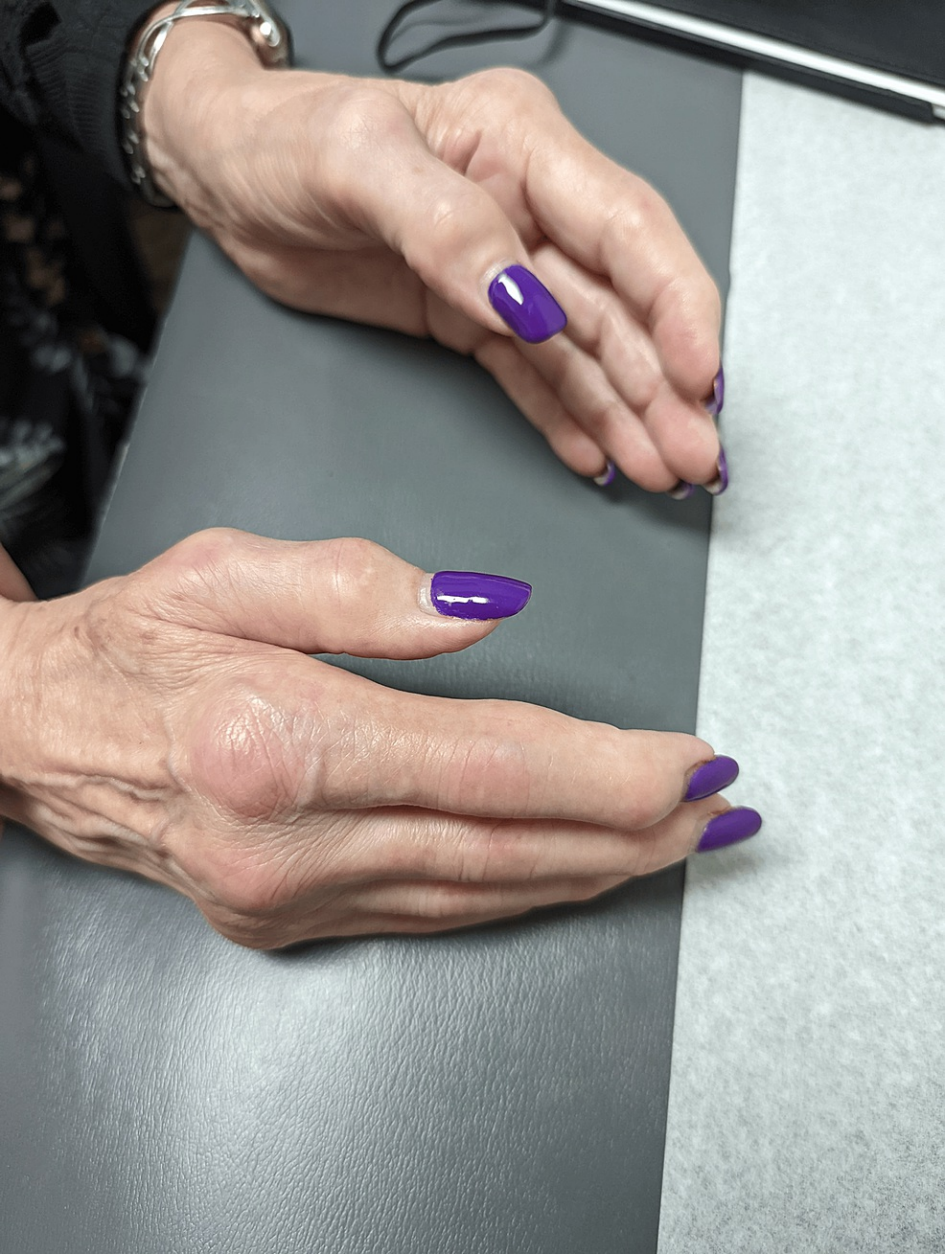
Palmar induration and thickening, characterized by firm, thickened skin, mild non-pitting edema, a rigid and hardened palmar surface, and MCP swelling MCP: metacarpophalangeal joint

**Figure 3:**
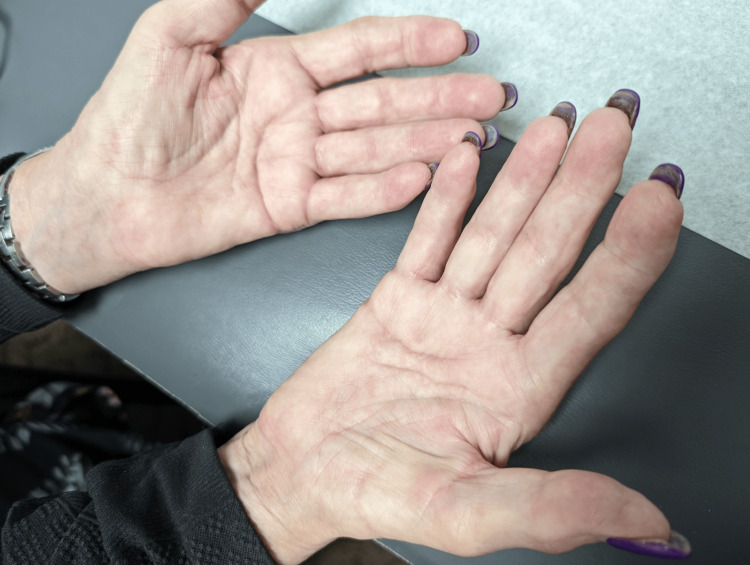
Palmar induration and thickening, characterized by firm, thickened skin, mild non-pitting edema, and a rigid and hardened palmar surface

**Figure 4:**
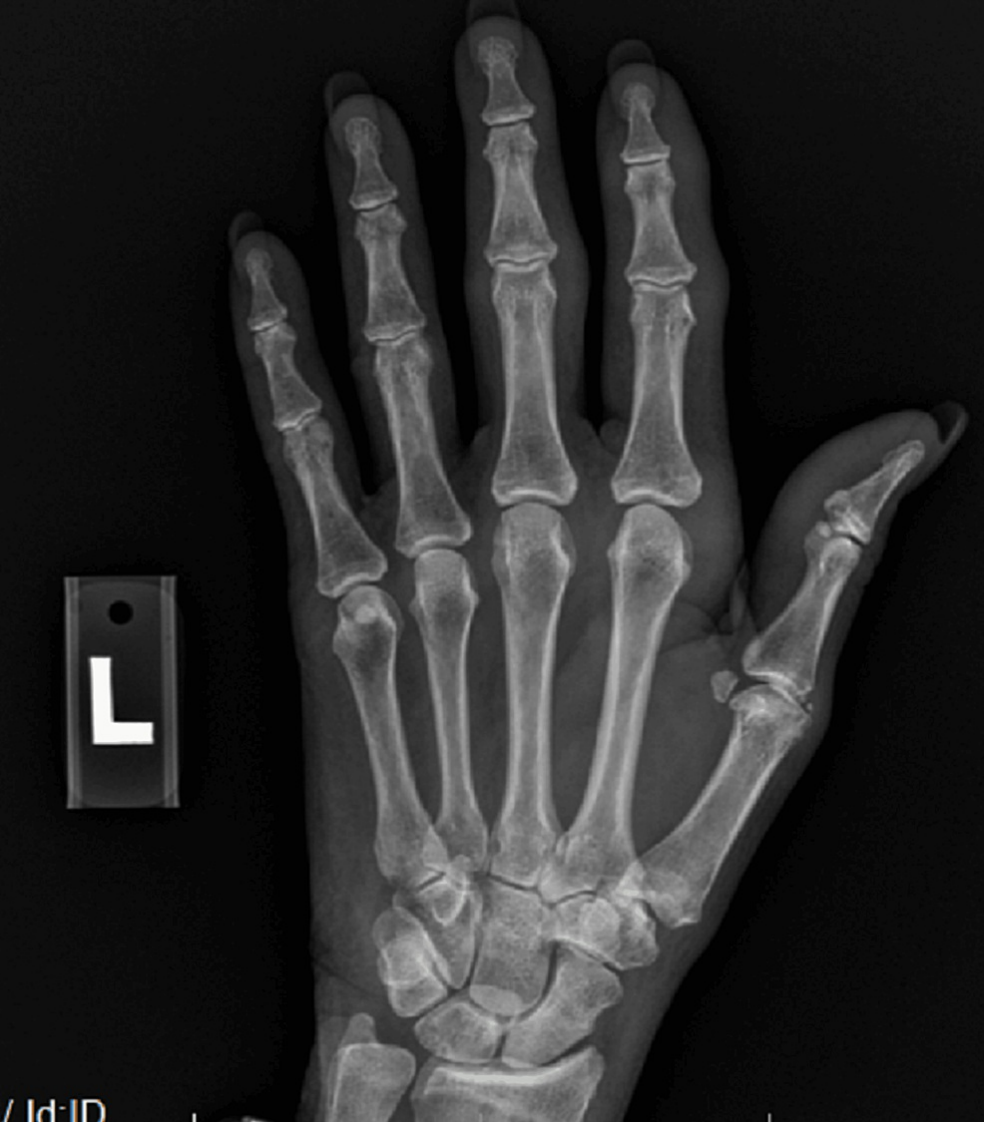
Radiographic examination of the hands indicates a reduction in joint space observed at the first CMC; PIPs, and DIPs​ CMC: carpometacarpal, PIP: proximal interphalangeal joint, DIP: distal interphalangeal joint

**Figure 5:**
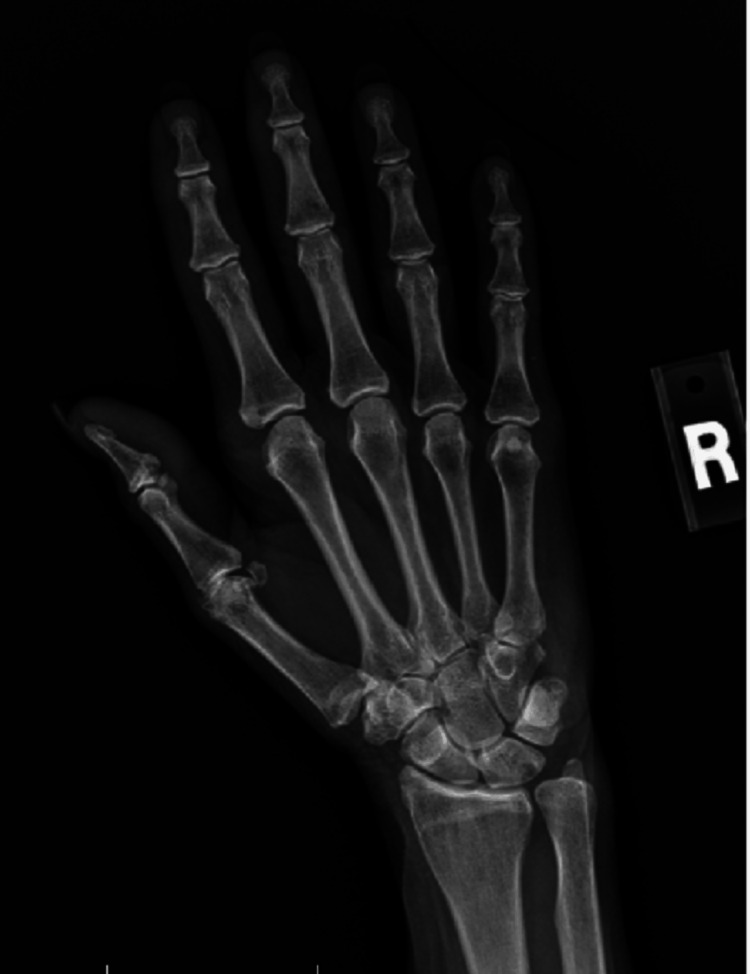
Radiographic examination of the hands indicates a reduction in joint space observed at the first CMC, PIPs, and DIPs​ CMC: carpometacarpal, PIP: proximal interphalangeal joint, DIP: distal interphalangeal joint

A 70-year-old Caucasian female initially presented with bilateral palmar paresthesia, hand pain, swelling, and pain in her distal interphalangeal (DIP), proximal interphalangeal (PIP) joints, and metacarpophalangeal joint (MCP) (Figures 1, 2) as well as her right second toe. Physical examination revealed skin thickening in her palms and fingers, nodular contractures, and reduced grip strength (Figure 3). Laboratory findings indicated an elevated speckled pattern ANA titer, high ferritin, and low hemoglobin levels while imaging revealed diminished joint spaces in the hands indicative of osteoarthritic changes (Figures 4, 5). Initial treatments, including non-steroidal anti-inflammatory drugs (NSAIDs), colchicine, meloxicam, Lyrica, and prednisone, offered minimal relief. Pain was moderately managed with moderate doses of tramadol, hydrocodone, and gabapentin.

The emergence of gastrointestinal symptoms led to pelvic CT with and without contrast months later, revealing a complex large anterior mass, fluid-filled endocervix, non-visible ovaries, midline cysts, adnexal nodules, and a sizable cervical mass. A diagnosis of stage 3C serous ovarian carcinoma was made, confirmed by CA125 positivity. The patient underwent a total hysterectomy, tumor debulking, and chemotherapy cycles with carboplatin, paclitaxel, and bevacizumab. She was then maintained on oral Olaparib and IV bevacizumab due to HRD positivity.

She achieved remission and reports improved outdoor activity capabilities with physical therapy and rehabilitation. This case underlines the intricacy of a patient with multi-systemic symptoms, found to have a major malignancy, and responding positively to targeted therapy.

## Discussion

Historically, PFPAS was called "woody hand" due to the appearance of hardened and indurated features. It is characterized by its disabling nature, which includes tightness, flexor contractures, and tethering of the affected area [3]. It was also called "shoulder-hand syndrome," a unique variant of reflex sympathetic dystrophy that involves severe, chronic limb pain, swelling, skin changes, and temperature variations. However, this name was less fitting for PFAPS because it differs in its rapid progression, bilateral involvement, and severity of symptoms, including the complete loss of function in affected extremities [4].

The underlying pathophysiology of PFPAS remains unclear. Explanations include activating specific pro-fibrotic factors by neoplastic cells or an autoimmune response involving antigens and antibodies within malignant cells. It has been attributed to connective tissue growth factor (CTGF), induced by TGF-β, to mediate stimulatory actions of TGF-β ECM and play roles in fibrosis pathogenesis. A skin biopsy of a contracture lesion would usually reveal significant fascia thickening and the presence of fibroblast spindle cells and collagen fibers [1,5]. Diagnosis starts with identifying its clinical features and associating it with age-related malignancy in this population and the use of tumor-associated markers (CA-125, CA 19-E4, CEA, alpha-fetoprotein (AFP), Inhibin, and PSA) for the culprit underlying malignancy.

In cases where musculoskeletal symptoms of PFAPS are considered idiopathic, a complete response is reported with corticosteroid treatment [6,7]. However, the extent of improvement can vary in cases with paraneoplastic symptoms linked to underlying malignancies. Addressing the underlying malignancy through methods like surgical excision or chemotherapy may result in variable enhancements, ranging from partial to complete [8]. It is noteworthy that in pioneering cases, consistent improvement was not observed, and unfortunately, all patients in those instances eventually succumbed to the condition [1].

## Conclusions

We report a case of PFPAS preceding serous ovarian cancer, emphasizing the need to view this syndrome as an early sign of paraneoplastic syndrome. Clinicians should screen for underlying malignancies when patients present with palmar fasciitis and non-inflammatory polyarthritis, which may indicate a rare condition with serious complications. Addressing the fundamental malignancy through methods like surgical excision or chemotherapy can yield varying degrees of improvement, ranging from partial to complete or no resolution. Further studies are required to investigate the pathophysiology, frequency, and association of PFPAS with various malignancies and recognition at an early stage. This also highlights the importance of interdisciplinary collaboration among rheumatologists, hand surgeons, dermatologists, oncologists, and gynecologists.
